# Cardiac hydatid disease; a systematic review

**DOI:** 10.1186/s12879-023-08576-3

**Published:** 2023-09-13

**Authors:** Erfan Banisefid, Kosar Baghernezhad, Rasa Beheshti, Sina Hamzehzadeh, Soheil Nemati, Zahra Samadifar, Hamid Owaysee Osquee, Elnaz Javanshir, Amirreza Naseri

**Affiliations:** 1 Student Research Committee, Tabriz University of Medical Sciences, Tabriz, Iran; 2 Cardiovascular Research Center, Tabriz University of Medical Sciences, Golgasht Street, Tabriz, East Azerbaijan, 5166/15731 Iran; 3 Department of Infectious Disease, Tabriz University of Medical Sciences, Tabriz, Iran; 4 Research Center for Evidence-Based Medicine, Iranian EBM Center: A Joanna Briggs Institute Center of Excellence, Tabriz University of Medical Sciences, Tabriz, Iran

**Keywords:** Echinococcosis, Cardiac Echinococcosis, Systematic review

## Abstract

**Background and objectives:**

Human cystic echinococcosis (CE), is a common health problem in low- and middle-income countries. Cardiac involvement is a relatively rare manifestation of *Echinococcus* infection. This study aims to summarize the evidence regarding the features of cardiac CE.

**Methods:**

Case series of the patients with cardiac CE, were included in this study. Non-English papers, case reports, reviews, letters, , commentaries, and conference abstracts were not included. A systematic search was conducted in PubMed and EMBASE databases and the risk of bias in the included studies was assessed using the Joanna Briggs Institute (JBI) Critical Appraisal Checklist.

**Results:**

Out of 3985 results of the searches, finally 37 studies were included in this systematic review. Based on available evidence, cardiac involvement is an uncommon but serious presentation of CE which presents with some non-specific signs and symptoms. Dyspnea, chest pain, and palpitation are the most common symptoms of the disease and normal sinus rhythm is the most common Electrocardiogram (ECG) feature. The disease is not associated with high mortality in case of timely diagnosis and appropriate management.

**Discussion:**

Consecutive and complete inclusion of participants, statistical analysis, and appropriate reporting of the demographics were the sources of bias in the included studies. The exclusion of non-English papers was a limitation during the review process.

**Funding:**

The research protocol was approved and supported by the Student Research Committee, Tabriz University of Medical Sciences (grant number: 69380).

**Registration:**

This study was registered in the International prospective register of systematic reviews (PROSPERO ID: CRD42022381204).

**Supplementary Information:**

The online version contains supplementary material available at 10.1186/s12879-023-08576-3.

## Introduction

Human cystic echinococcosis (CE), also known as hydatid disease, is a tissue infestation, which is endemic in many sheep-farming areas of the world, notably Mediterranean countries, the Middle East, South America, and Australia [1, 2]. Echinococcosis is a common health problem in low- and middle-income countries [3], and the European Registry of CE found it a neglected health problem in these countries [4]. CE has a considerable economic impact, with the cost of the disease approximated at 0.03% of the country’s gross domestic product in Iran [5]. CE occurs mainly as a result of infection with the larvae of *Echinococcus granulosus*. Most often, dogs and other carnivores are the primary hosts and sheep are intermediate hosts, whereas humans are accidental hosts of the parasite. Humans usually become infected by ingesting food, milk, or water contaminated by dog feces containing the ova of the parasite [6, 7]. The most common sites of CE involvement are the liver and lung [8]; however, it can affect other organs in the body such as the heart, too [9]. Cardiac CE is a relatively rare manifestation of *Echinococcus* infection, which represents 0.5 to 2% of CE cases [10–12] Despite the multiple studies regarding the features of cardiac CE [13, 14], there was no systematic review of the literature on this topic, therefore this study aimed to determine the characteristics of cardiac CE disease based on available evidence.

## Methods

This study was conducted following the Preferred Reporting Items for Systematic Reviews and Meta-Analysis (PRISMA) 2020 statement [15].

### Eligibility criteria

Case-series studies which presented the features of cardiac CE were considered for inclusion in this systematic review. Non-English studies, case reports, conference abstracts, review articles, letters, and commentaries were excluded.

### Literature search

Medline via PubMed and EMBASE databases were searched systematically from inception to March 2022 with the following keywords: ((Echinococc* OR Hydatid*) AND (Heart OR cardi*)) OR Cardiac Hydatid in title, abstract, and author keywords. During the search, no restrictions on language and type of study were applied. Also, forward and backward searches from references and cited studies of included studies were performed for a comprehensive coverage of the published studies.

### Study selection and data collection

Two authors separately reviewed the full-texts of the studies after being primarily screened based on title/abstract. The data from the included studies were extracted into a Microsoft Excel table. Information from eligible studies, including the name of the first author, publication year, study setting, patients’ demographics, the site of the heart’s involvement, the involvement of other organs, clinical manifestations, Electrocardiogram (ECG) findings, echocardiography, Chest X-ray (CXR) or magnetic resonance imaging, laboratory findings, management, final outcome, and follow-up, is extracted by two authors and cross-checked. Disagreements between authors were solved by the third author.

### Risk of bias assessment

The potential for risk of bias in the studies was assessed by The Joanna Briggs Institute (JBI) Critical Appraisal tool for Case Series. The validity and reliability of condition evaluation, the contiguous recruitment of participants, the reporting of patient demographic and clinical data, clinical management, and follow-up are the sources of bias that being evaluated by this checklist.

## Results

### Search and selection process

**Fig. 1:**
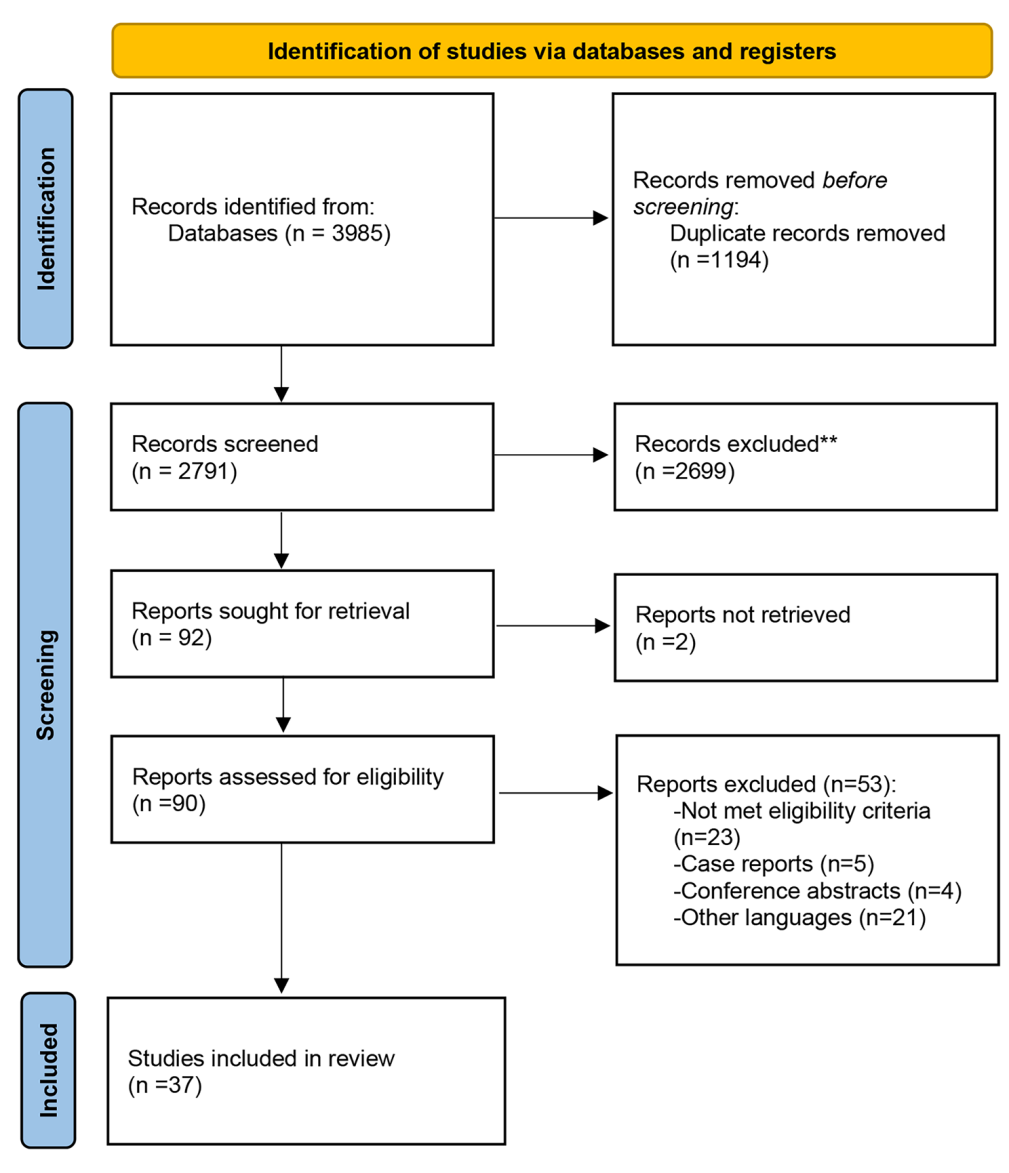
PRISMA 2020 flow diagram

Electronic searches in the databases results in 3985 records and finally, 37 studies met our criteria for inclusion in this systematic review. The details of the selection process are presented in Fig. 1.

### Study characteristics

Seventeen studies were conducted in Turkey and the setting in other studies were Tunisia (5 studies), Iraq (3 studies), Spain, Syria, China, Greece (2 studies for each), Saudi Arabia, Kyrgyzstan, India, and France (1 study for each). The sample of cardiac CE in the studies varies between 2 and 62.

### Risk of bias in studies

**Fig. 2:**
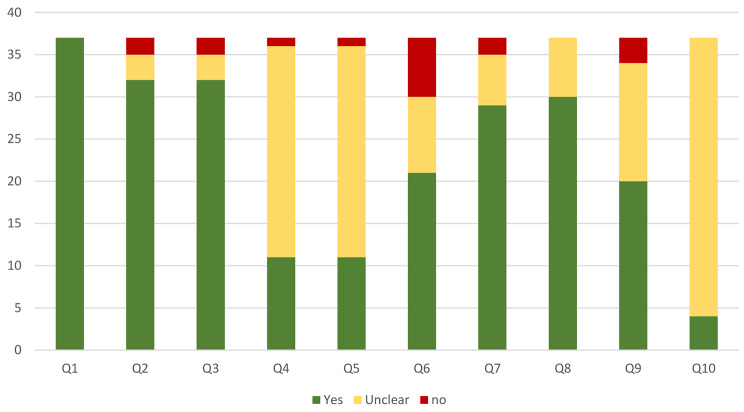
Summary of risk of bias assessments based on The Joanna Briggs Institute Critical Appraisal tool for Case Series *Q1: Were there clear criteria for inclusion in the case series?* *Q2: Was the condition measured in a standard, reliable way for all participants included in the case series?* *Q3: Were valid methods used for identification of the condition for all participants included in the case series?* *Q4: Did the case series have consecutive inclusion of participants?* *Q5: Did the case series have complete inclusion of participants?* *Q6: Was there clear reporting of the demographics of the participants in the study?* *Q7: Was there clear reporting of clinical information of the participants?* *Q8: Were the outcomes or follow up results of cases clearly reported?* *Q9: Was there clear reporting of the presenting site(s)/clinic(s) demographic information?* *Q10: Was statistical analysis appropriate?*

Figure 2 is a summary of the risk of bias assessments based on The JBI Critical Appraisal tool for Case Series. The details are reported in supplementary material 1. Based on our assessments, the most common possible sources of bias in the included studies were regarding the consecutive and complete inclusion of participants and statistical analysis, which were not reported in a clear form for a conclusive judgment. Inappropriate reporting of the demographics was also another source of bias in the studies.

### Results of individual studies

The evidence regarding the site of involvement, other organ involvement, signs and symptoms, ECG findings, echocardiography or CXR findings, laboratory findings, management, outcome, and follow-up are summarized and presented in Table 1. Cardiac CE can infect people of any age in both males and females. LV is reported as the most common site of infection. Studies suggested that cardiac CE can be secondary to other site infections, such as lung, liver, and spleen; however, cases of cardiac involvement without other organ involvement are also documented. The most commonly reported symptoms were chest pain, dyspnea, and palpitation and normal sinus rhythm (NSR) was the most common declared ECG feature. Regarding the laboratory findings, serological tests such as echinococcus indirect hemagglutination (EIHA) and enzyme-linked immunosorbent assay (ELISA), as well as eosinophilia, and positive casoni were the most commonly reported findings. A combination of surgical methods, such as cardiopulmonary bypass, and pharmacological management were reported in the studies; which were associated with appropriate outcomes, so the rate of mortality and recurrence were not considerable.

**Table 1:** – summary of included studies

Study	Setting (Hospital, City, Country)	Number of cases	Male/ Female	Age	Site of involvement in heart	Other organs involvement	Signs and symptoms	ECG findings	Echocardiography or CXR findings	Laboratory findings	Management	Final outcome and follow-up
Alptekin Yasim (2017) [17]	Southeast Anatolian, Turkey	25	15/ 10	33.4 ± 12.6 (15–75)	Intracavitary (11) including: IAS (3), IVS (2), RV (2), RA (2), RV outflow tract (1), mitral valve posterior annulus (1), extra-cavitary (14) including: LV wall (10), RV wall (1), Multiple cysts in the LV lateral wall and apex (1), LV wall and left atrial outer wall (2)	lung (4), liver (4)	Dyspnea (18), Palpitation (7), Chest pain (7), Fatigue (2) Left arm pain (1)	NSR (13), nonspecific ST-T changes and T-wave negativity (11), RBBB (1)	-	WBC: 9.716 ± 1.855 Neutrophil: 62.7% ± 8.2% Lymphocyte: 26.1% ± 7.2% Eosinophil: 2.8% ± 3.1% ESR: 34.4 ± 13.7 CRP: 17.6 ± 8.5 EIHA + (23)	CPB (24); Albendazole for 28 days for four additional cycles of 28 days each with 14 days between each 28-daycycle.	*Mortality due to hepatic failure two years after the surgery (1)*
von Sinner (1995) [40]	Saudi Arabia	15	8/ 7	(15–56)	in 4 primary involvements; LV /pericardium (1), IVS /pericardium (1), LA/pericardium/mitral valve (1), RA/pericardium/tricuspid valve (1)	mediastinum (3), lung parenchyma (6), extension from the liver and abdomen (2)	-	-	-	-	-	-
Omac Tufekcioglu (2007) [24]	Turkey	16	7/9	41 ± 18.3	Pericardium: LV lateral wall (3), RV free wall (1), posterior of RA (1), RV apex (1), RV free wall (1) Myocardium: LV lateral wall (3), RV apex (2), LV septum (1), LV aspect of septum (1), LV apex (1), RV outlet (1), LV posterior wall (1) Anterior ascending aorta (1)	Liver (5), Spleen (3), Lung (3), Brain (1)	Dyspnea (5), Palpitations (4), Angina (3), Ascites and pretibial edema (1), none (3)	LBBB (3), RBBB (2), NSR (11)	Increased cardiothoracic ratio (6)	serology + (16)	Surgical resection and albendazole (16)	No recurrence or complications
Ali Gurbuz (2003) [41]	Ataturk Hospital, Yeilyurt, Izmir, Turkey.	6	1/5	40 ± 5	RV outflow tract (2), LV outflow tract (1), RA (1), the RV (1), the right atrioventricular groove (1)	Liver (2), Spleen (1)	Dyspnea (6), chest pain (3), dizziness (1), palpitation (2)	NSR (3), Ventricular premature beat (1), non-specific ST changes (1), V3-6 negative T waves (1)	Myxomatous pedunculated mass adhered to IAS (1)	EIHA + (5)	Stemotomy, CPB mild hypothermia (28° C) (5)	ventricular septal defect after 2 years (1)
Cemal Levent Birincioğlu (2003) [42]	Turkey.	10	7/3	-	LV cavity (5), RV (3), RA (1), Intrapericardial (1)	Lung (1), Spleen (1)	Dyspnea (8), Syncope (3), Vomiting (1), chest pain (6), palpitation (3), fever (1)	NSR (5), Sinus tachycardia (2), D1-3, V4-6: T (-) V5-6: Q (1), V4-6, D1-aVL: T (-) (1), V1-3: T (-), ST increased (1)	daughter cysts neighboring the RA (1)	Eosinophilia (1) EIHA + (8)	benzimidazole derivatives (preoperatively and postoperatively) (10), albendazole for 5 years postoperatively (1) operation without CPB (10)	Reoperation 68 months after first operation due to reoccurrence (1)
Marta Diaz-Menendez (2012) [43]	Ramón y Cajal Hospital, Madrid, Spain.	11	7/4	- 49.4	RA (6), IVS (2), LA and pericardium (2), RV (1), IVC involvement (5)	Lung (7), Liver (7), Kidney (2), Spleen (2)	chest pain (7), dyspnea (4), hemoptysis (2), no symptoms (2)	atrial hypertrophy (1), LBBB (1), RBBB (1)	-	Eosinophilia (400 cells/mm^3^) (6) EIHA + (11)	CPB (9), albendazole 15 mg/Kg/d (9) praziquantel 40 mg/Kg/d (6) for average of 37 months	AF responsive to antiarrhythmic drugs (1) Mortality during surgery duo to rupture of the cyst in IVC (1)
Rüçhan Akar (2003) [37]	Ankara, Turkey	62	23/39	-	LV (29), IVS (12), RV (12), RA (7), LA (1), Sinus of Valsalva (1)	lung, liver, brain, and peritoneal, and/or renal hydatid cysts (12)	Dyspnea (39), Palpitation (25), Chest Pain (14), Syncope (3), Cough (8), Hepatomegaly (7), Hemoptysis (6), Pulmonary embolism (4), Acute abdomen (2), Peripheral embolism (3), Asymptomatic (3), Cyanosis (2), Cerebral embolism (2), Constrictive Pericarditis (1), Anaphylactic reaction (1), Cardiac tumor (1)	AF (6), RBBB (5)	-	Casoni + (11) EIHA + (42)	Thoracotomy with CPB (50)	Mortality duo to pulmonary embolism (2), Mortality duo to rupture of IVS (1)
Jaffar Shehatha (2009) [44]	Ibn-Alnafis Hospital, Baghdad, Iraq	4	-	33.75	LV (4)	Liver (3), Lung (1)	Dyspnea (4), palpitation (4), easy fatigability (4)	-	pericystic layer consisted of fibrous tissue (4)	Casoni + (4)	Thoracotomy with CPB (4), Albendazole after surgery (4)	No recurrence or complications
Eylem Tuncer (2010) [45]	s	13	7/6	36 ± 18.3	RA base (1), LV anterior wall (1) LA posterior wall (1), IVS (2), LV anterolateral wall (1), IAS (1), RV mural anterior wall (1), LV mural anterior (1), RV mural wall (1), RV inferior mural wall (1), LV intracavitary apical wall (1), LV mural apical wall (1)	Liver (5) Lung (3) Brain (1) Kidney (1)	Dyspnea (5), Fatigue (2), Palpitation (3), Fever (1), Cough (1), Chest pain (3), Syncope (1)	NSR (13)	-	-	Thoracotomy with CPB (13)	complete atrioventricular block (necessitated pacemaker) (1), reoperation for recurrent cardiac hydatidosis, one year after the initial surgery (1)
A. Abid (1991) [31]	Lu Rabta Hospital, Tunis, Tunisia	6	2/4	22	LV (3), Multiple cysts rupturing in pericardium (3)	-	-	NSR (4), ST changes (2)	pericardial effusion (2)	-	Thoracotomy with CPB (6)	Septicemia due to Enterobacter (1)
Djoshibaev (2003) [33]	Bishkek, Kyrgyzstan	5	2/3	18	pericardial and epicardial involvement (1), apicolateral wall of the LV (1), IVS (3)	None	Neck vein distention (1), hepatomegaly (3), hypotension (1), fever (1), Palpitations (1), Oedema (2), Dyspnea (2), Weakness (2)	-	pericardial effusion (1)	-	Thoracotomy with CPB (3), Surgery on a beating heart (2), Albendazole for 2 years after surgery (5)	No recurrence or complications
Tall Bashour (1996) [46]	Aleppo, Syria	19	13/6	48	LV cavity (10), RV cavity (6), Pericardial (2)	-	Chest pain (19), dyspnea (11), palpitation (8), fever (1)	T inversions (15), ST depression (7), premature ventricular beats (8), incomplete or complete BBB (11) supraventricular tachycardia (2)	-	Casoni + (9)	-	-
Khaldoun Ben-Hamda (2003) [47]	Fattouma Bourguiba Hospital, Monastir, Tunisia	14	7/7	27.7	Pericardium (3), LV anterior wall (3), IVS (4), RV (2), LV apex (1)	Liver (4), Lung (5), Spleen (1)	Chest pain (6), Dyspnea (2), systolic murmur in pulmonary area (3), Pulmonary hypertension (1)	Inverted T waves (6), RBBB (2), NSR (5), RV hypertrophy (2)	-	EIHA + (13)	-	-
Birincioglu (2013) [48]	Turkey	41	19/22	36	Lateral LV (5), Pericardium (14), Apex (9), Septum (9), Posterolateral LV (9), Anterior RV (8), Anterolateral LV (6), Inferior LV (7), Behind aorta (1), RV outflow tract (1), behind pulmonary vein (1), Between RA and aortic sinus of Valsalva (3)	Lung (9), Liver (6), Spleen (3), Brain (2), lumbosacral vertebrae (1), kidney (1)	dyspnea (23), chest pain (19) palpitations (13) syncope (6) weakness (4)	-	-	EIHA + (27)	Albendazole 15 mg/kg 2 weeks before and 12 weeks after surgery median sternotomy (40) posterolateral thoracotomy (1)	Mortality in the postoperative period duo to hemodynamic collapse after rupture of the cyst (1), relapse (1)
Hatem Bouraoui (2005) [49]	Farhat Hached Hospital, Sousse, Tunisia	12	2/10	40	LV (3), RV (3), RA (3), pericardium (3)	Lung (2)	chest pain (7), dyspnea by exertion (4), palpitation (1)	T wave inversion (8), ST depression (5), incomplete RBBB (2), AF (1)	intracystic trabeculation (4), a cystic mass with a central nucleus and some small echolucent areas (4), The spherical mass (3), 15 mm echogene mass attached to the septal tricuspid valve (1)	eosinophilia (7) serology + (6)	-	-
Vedat Erentug (2004) [50]	Kosuyolu Heart and Research Hospital, Istanbul, Turkey	6	2/4	20–68	RV anterior wall (1), IVS (2), LV posterior wall (1), RV apical (1), RV inferior wall (1)	Lung (2), Liver (3), Brain (1), Kidney (1)	dyspnea (2), Palpitation (2), Asymptomatic (4)	-	-	-	Thoracotomy with CPB (6) Albendazole 400 mg/day (6)	No recurrence or complications.
Fei Yan (2015) [51]	Xinjiang, China	26	15/11	28.9 ± 7.6	LV (7), RV (5), RA (2), IVS (1), Pericardium (6), Multiple (5)	Liver (3), Lung (3), Lung and liver (1)	Asymptomatic (3), Dyspnea (15), Chest pain (11), Palpitation (8), Cough (7), Fever (2)	NSR (7), LVH (5), RVH (2), incomplete RBBB (3), incomplete LBBB (1), ST changes (8)	-	Eosinophilia (15), Casoni + (15), EIHA + (15)	Thoracotomy with CPB (26), puncture–aspiration cystectomy, intact endocyst enucleation and total cyst resection. (26), Albendazole after surgery (26)	No recurrence or complications
Niyazi Gormus (2004) [52]	Konya, Turkey.	7	2/5	37	Pericardium and RV (2), LV (2), RA (1), LA (1), RV outlet (1)	None.	Pulmonary emboli (1), Asymptomatic (6)	-	-	EIHA + (7)	CPB cystectomy Capitonnage (7) Thoracotomy with CPB (7), Mebendazole 15 mg/kg for 2 years after surgery	No recurrence or complications
Vivek Wadhawa (2018) [32]	Gujarat, India	10	6/4	35.9 ± 12.04	LV anterolateral wall (2), LV mural wall (1), LV, LA, IVS (1), Pericardial cyst and RV mural anterior wall (1), Pericardium and LV posterolateral wall (1), LV posterolateral wall (1), IVS (1)	Lung (1) Kidney (1)	Dyspnea (7), Chest pain (7), Palpitation (1), Cough (1), Asymptomatic (1), Fatigue (6)	-	moderate pericardial effusion (1)	EIHA + (5), Casoni + (2)	Thoracotomy with CPB (9), cystectomy and partial pericardiectomy without CPB (1), albendazole 400 mg BID for 12 weeks after surgery (10)	No recurrence or complications
Marah Jamli (2020) [53]	Sahloul Hospital, Sousse, Tunisia	27	Male predominance (sex ratio of 1.7)	35	LV (5) RV (7) LA (3) RA (2) Pulmonary artery (2) SVC (1) Inferior vena cava (1) Right inferior pulmonary vein (1) Septal (5) Ventricular septum (3) Atrial septum (2)	-	Nontypical chest pain (17), Dyspnea (10), Palpitations (16), Hemoptysis (5), Syncope (2), Lipothymia (3), Cough (6), Constrictive chest pain (1), Asymptomatic (8)	T inversion (15) LBBB (4) RBBB (2) Atrioventricular block (2)	-	Serology + (10)	Thoracotomy with CPB (27)	mortality duo to severe acute cardiac failure at postoperative hour 17 (1), mortality duo to multiorgan failure on postoperative day 8 (1)
Sami S Kabbani (2007) [54]	Damascus, Syria	19	8/11	25.6 ± 12.8	RA (1), LA (1), LV anterior wall (2), LV posterior wall (2), LV cardiac apex (2), RV anterior wall extending to tricuspid annulus (1), RV outflow (1), IVS (6), IVS combined with LV epicardial cyst (1), Pericardium (2)	Lung (4) Liver (2) Kidney (1)	dyspnea (9), Chest pain (6), Cough (6), Hemoptysis (5), Palpitations (4), Abdominal pain (4), Dysphagia (1), Anaphylactic shock (2), Skin rash (2), Seizures (2), Cerebrovascular accident (1)	LVH (2) RVH (1) Incomplete RBBB (6) Incomplete LBBB (2) nonspecific c ST/T changes (8)	-	EIHA + (12) elevated ESR (10) eosinophilia (6)	Thoracotomy with CPB (19)	Mortality due to cerebral hydatidosis 2 years postoperatively (1), Mortality duo to multiorgan failure after the surgery (1)
Mehmet Kaplan (2001) [55]	Siyami Ersek, Istanbul, Turkey	8	1/7	33.6 ± 14.3	RV outlet (2), RV apex (1), LV apex (1), LA free wall (1), RV (1), LV (1), Muscular septum right side (1)	Liver (1)	Dyspnea (8) Palpitation (7)	-	-	-	Thoracotomy with CPB (8) Mebendazole 250 mg BID 6 months after surgery (8)	No recurrence or complications
F. Kardaras (1996) [38]	Evangelismos General Hospital, Athens, Greece.	10	6/4	46.5	LV (4), RA (1), IVS (1), IAS (1), Pericardium (1), RV (1), Multiple myocardium cysts (1)	-	Asymptomatic (2) Chest pain (5) Anaphylactic episodes (2) CHF (1) Pericarditis (1) Dyspnea (1) Multiple atrial emboli (1)	-	-	Casoni + (4) ELISA + (4) Weiberg (4) EIHA + (4)	-	Mortality duo rupture of pulmonary echinococcal cyst (1), Mortality duo massive pulmonary hydatid embolism (1), Mortality duo rupture of an undiagnosed hydatid cyst of the RA during cannulation for CPB (1), Recurrent systemic embolism and became hemiplegic (1)
A. Miralles (1994) [56]	La Pitie Hospital, Paris, France.	8	3/5	27 ± 17	LV wall (7), RV wall (1)	Liver (5), Lung (3), Kidney (1), Costal (1)	Asymptomatic (4) Cough (3), Chest pain (4), Pain (4), Fever (1) Hemoptysis (1), Dyspnea (2), Cerebral accident (1)	-	-	-	Thoracotomy with CPB (8)	atrioventricular block with a cyst invading the IVS (1)
Vakeli Murat (2007) [57]	Urumqi, China.	15	9/6	23.0 ± 8.5	RA (3), left anterior myocardium (7), pericardium (5)	Not involved	-	-	-	Serology + (12)	Thoracotomy with CPB (8) Thoracotomy without CPB (7) mebendazole 50 mg/kg orally 4-times daily for 3 months (15)	recurrent cysts (4)
Omer Tanyeli (2017) [58]	Konya, Turkey.	12	6/6	42.6	RA (2), RV (2), RV outlet (1), LV (5), LA (1), IVS (1)	-	-	-	-	-	Thoracotomy with CPB (10), Thoracotomy without CPB (1) Left AL thoracotomy without CPB (1) Mebendazole (6) Albendazole (6) 400 mg bid	No recurrence or complications
Kutay Tasdemir (2000) [59]	Kayseri, Turkey	2	2/0	18	LV (1), IVS (1)	-	Dyspnea and syncope (1) Extremity ischemia (1)	-	-	-	surgical resection (2)	No recurrence or complications
Ertan Onursal (2001) [60]	Istanbul Medical Faculty, Istanbul, Turkey.	8	4/4	33.5	IVS (2), RA (1), Pericardium (5)	Liver (4), Lung (3), Brain (1)	Chest pain (5), Cough (3), Dyspnea (4), Fever (3), Palpitation (3), Abdominal pain (1), Syncope (1), Acute respiratory distress (1), Headache (1), Vomiting (1)	nonspecific ST wave changes (3), premature ventricular beats (1), supra ventricular tachycardia (1)	-	-	Thoracotomy with CPB (2) Thoracotomy without CPB (6)	mortality duo septic shock on the 23rd postoperative day (1)
Kutay Tasdemir (2009) [61]	Kayseri, Turkey.	10	8/2	27	RV (2), LV (5), RA (1), LA (1), Descending aorta, abdominal aorta (1)	Lung (1)	Pain (3), pallor (3), pulselessness (3), paresthesia (3), paralysis of lower limb (3), dyspnea (1), Palpitation (1)	-	-	-	Thoracotomy with CPB (8) Embolectomy (3) Albendazole 400 mg BID for 2 months after surgery (10)	mortality because of acute renal insufficiency after 48 h of the first operation (1), mortality duo a ruptured cyst while operating (1)
Haitham Noaman (2017) [20]	Ibn Al-Bitar Hospital, Bagdad, Iraq.	19	8/11		LV (9), pericardium (5), RV (3), IVS (2)	Lung (1)	chest heaviness (2), chest pain (2), dyspnea (2), palpitations arrhythmias (2)	-	left-sided heart failure and mitral valve dysfunction (9), RV outlet obstruction with right-sided heart failure (3) arrhythmias (2)	-	open-heart surgery (19)	lost to follow-up (13), no recurrence or complications (6)
Jose M. Oliver (1988) [62]	Madrid, Spain.	13	8/5		Pericardium (3), LA wall (1), LV (3), RV (2), inter ventricular septum (5)	Lung (8), Liver (2), Brain (2), Kidney (2)	Dyspnea (1), Chest pain (4), Anaphylaxis (2), Pulmonary hypertension (2)	Nonspecific ECG changes (4)	Cardiomegaly (1), Tricuspid stenosis (1), Tricuspid regurgitation (1), LV, RV protrusion Pericardial effusion (1), SVC syndrome (1)	-	-	-
Ashur Y. Oraha (2018) [63]	Kurdistan, Iraq.	4	2/2		pericardium (1), IVS (1), Apex (1), LV (1)	Liver, spleen (1) lung (2)	nausea and vomiting (1), Fever (1), chest pain (2), Strider (1), Palpitations (2), Dyspnea (1)	-	-	-	median sternotomy and CPB	-
A.Abid (2003) [64]	La Rabta Hospital, Tunis, Tunisia	7	3/4	19 ± 4	RV (3), IVS (4), RA (2)	Lung (6)	Palpitations (2), Chest pain (2), Hemoptysis (2), dyspnea (1),	NSR (3), ST modifications (3), T wave < 0 (3), Ventricular Extrasystoles (1)		Serology + (4)	Right ventriculotomy (1), Right auriculotomy (6)	Satisfactory (5) Right pneumothorax (1) Left pneumothorax (1)
Özge Altaş (2014) [65]	Sakarya, Turkey.	3	3/0	38	IVS (2), LV (1)	Liver (1)	Dyspnea (2) Chest pain (1) Fatigue (1)	T wave changes in inferior Leads (2)	a fluid content with a homogeneously hypointense signal intensity on T1-weighted images and a homogeneously hyperintense signal on T2-weighted images	-	elective operation through median sternotomy, and CPB	No recurrence or complications
Dursun Atilgan (2002) [66]	Istanbul, Turkey.	3	1/2	39.25	RA (1), IVS (1), LV (1)	-	Palpitation (2), dyspnea (2), Fatigue (1), Cough (1)	inverted T waves	-	-	albendazole digoxin, furosemide, spironolactone surgery	No recurrence or complications
John Barbetseas (2005) [18]	Hippokration Hospital, Athens, Greece.	3	1/2	31	LV (2) IVS (1) pericardium (1)	Liver (1)	Palpitation (1), anaphylactic symptoms (1), left-sided hemiplegia (1)	first-degree atrioventricular Block, T-wave inversion	-	Serology + (2)	Surgery albendazole	No recurrence or complications
Nazim Kankilic (2020) [19]	Şanlıurfa, Turkey.	4	0/4	23	LV (2) RA (1) RV (1)	Lung (3) Liver (1) Brain (1)	Palpitation (1), chest pain (1), Dyspnea (4), Headache (1), abdominal pain and abdominal swelling (1)	-	-	-	Normothermia + CPB + cystectomy	-

ECG: electrocardiogram; WBC: White Blood Cell; LV: left ventricle; RV: right ventricle; RA: right atrium; IVS: interventricular septum; IAS: interatrial septum; RBBB: Right bundle branch block; LBBB: Left bundle branch block; NSR: Normal sinus rhythm; AF: Atrial fibrillation; SVC: Superior vena cava; ELISA: enzyme-linked immunosorbent assay; EIHA: echinococcus indirect hemagglutination; CPB: Cardiopulmonary bypass; ESR: Erythrocyte Sedimentation Rate;

## Discussion

This study is the first systematic review to summarize the evidence regarding the features of cardiac CE. Cardiac involvement occurs mainly by invasion of myocardium from the coronary arteries or pulmonary veins as a result of rupture of pulmonary echinococcal cysts [16]. Various locations of the heart can be infected with CE; however, LV is the most common site [17–22] and it can be presented in the pericardium as well as the myocardium layer, too [23, 24]. The main reason for higher rate of LV involvement can be the dominance of the left coronary artery [13, 25].

The clinical presentation of CE ranges from asymptomatic to life-threatening conditions and sudden death. Symptoms depend on location, size, compression, or involvement of abutting structures. The most frequent symptoms of CE are dyspnea, chest pain, and palpitation. The stretch of pericardium and/or compression of coronary vessels can be an explanation for this [11, 26, 27]. Also, the NSR is the most common ECG finding in CE patients, which suggested a crucial role of imaging and laboratory methods in the detection of cardiac CE.

Cardiac CE can affect almost everyone in each age group in the endemic areas; however, non-specific signs and ECG changes, make it a diagnostic challenge. Transthoracic echocardiogram (TTE) is suggested to be sensitive and specific in the diagnosis of cardiac CE [28, 29]. Classic cystic CE lesions appear as well-defined echo-lucent structures on echocardiographic images, whereas those that have become solidified, calcified, or degenerated can be hyper-echogenic and, thus, mimic semi-solid or solid mass lesions [30]. In a case series 6/16 (37.5%) of increased cardiothoracic ratio was reported [24]. Also, 5 cases of pericardial effusion were reported in the included studies [31–33]; which suggested that these features can be sign of cardiac CE, too. Cardiac CE infection is predominantly secondary to more commonly involved organs such as the liver and lung [34], therefore, in patients with CE in other organs, imaging and blood tests can help detect the cardiac form of the disease. Serological methods such as EIHA and ELISA are suggested as highly sensitive and specific tests for CE [14, 35]; however, the diagnostic accuracy of these blood tests, specifically in the cardiac form of the disease is not investigated. Most of the included studies reported a considerable rate of positive EIHA in patients with cardia CE; however, there is a need for future diagnostic accuracy studies of EIHA in cardiac CE, for clinical recommendation.

Cardiac CE is an uncommon but serious condition; however, it is not associated with a high rate of mortality in case of timely diagnosis and appropriate surgical and non-surgical management [36]. Overall, there was no significant cardiac CE-related mortality in the included studies. Rupture of the cyst during surgery, pulmonary embolism, and multi-organ failure was the cause of mortality in cardiac CE patients [17, 37, 38]. Also, pharmacotherapy with mebendazole or albendazole for 6–24 months postoperatively, is recommended for prevention of the recurrence of the disease [39].

Systematic and predefined approach of the review and a comprehensive database search as well as hand searching for full coverage of the published evidence were the main strengths of this study. The exclusion of non-English papers was the main limitation of this study. Future multi-center studies with larger sample sizes and comprehensive reports of the characteristics of the disease can strengthen the evidence in this regard and help appropriate management in cardiac CE patients. Also, it is recommended to report more details of cardiac CE patients in future studies.

## Conclusion

Cardiac involvement is an uncommon presentation of CE which is associated with some non-specific signs and symptoms such as dyspnea, chest pain, palpitation, and fatigue. In the case of diagnosed CE in other organs such as the lungs and liver, cardiac CE, should be considered by clinicians. Non-specific signs and symptoms make cardiac CE a diagnostic challenge; however, imaging methods such as TTE as well as serological tests, can help diagnosis of the disease. Cardiac CE is not associated with considerable mortality in the case of timely diagnosis and appropriate management.

### Electronic supplementary material

Below is the link to the electronic supplementary material.

Supplementary Material 1

## Data Availability

All data generated during this study are included in this published article and its supplementary information file.
