# Supplementary material for: Cardiac hydatid disease; a systematic review
Source: BMC Infect Dis. 2023 Sep 13;23:600. doi: 10.1186/s12879-023-08576-3 (PMC10500901; doi:10.1186/s12879-023-08576-3)
Supplement: Supplementary file 1 — Supplementary Material 1 [file 12879_2023_8576_MOESM1_ESM.docx]

**Supplementary table 1 – details of risk of bias assessments based on The Joanna Briggs Institute Critical Appraisal tool for Case Series.**

| **Study** | **Q_1_** | **Q_2_** | **Q_3_** | **Q_4_** | **Q_5_** | **Q_6_** | **Q_7_** | **Q_8_** | **Q_9_** | **Q_10_** |
| --- | --- | --- | --- | --- | --- | --- | --- | --- | --- | --- |
| Alptekin Yesim (2015) [1] | Y | Y | Y | Y | Y | Y | Y | Y | Y | U |
| von Sinner (1994) [2] | Y | Y | Y | N | N | U | U | U | N | U |
| Omac Tufekcioglu (2007) [3] | Y | Y | Y | Y | Y | Y | Y | Y | Y | U |
| Ali Gurbuz (2003) [4] | Y | Y | Y | U | U | Y | Y | Y | Y | U |
| Cemal Levent Birincioğlu (2002) [5] | Y | Y | Y | U | U | Y | Y | Y | Y | U |
| Marta Diaz-Menendez (2012) [6] | Y | Y | Y | U | U | Y | Y | Y | Y | Y |
| Rüçhan Akar (2003) [7] | Y | Y | Y | Y | Y | N | N | Y | N | U |
| Jaffar Shehatha (2009) [8] | Y | U | U | Y | Y | N | U | Y | Y | U |
| Eylem Tuncer (2010) [9] | Y | U | U | U | U | Y | Y | Y | Y | U |
| A. Abid (1991) [10] | Y | Y | Y | U | U | N | U | Y | U | U |
| Djoshibaev (2001) [11] | Y | Y | Y | Y | Y | Y | Y | Y | U | U |
| Tall Bashour (1996) [12] | Y | Y | Y | U | U | N | U | U | Y | U |
| Khaldoun Ben-Hamda (2002) [13] | Y | Y | Y | Y | Y | Y | Y | U | U | U |
| Birincioglu (2012) [14] | Y | Y | Y | U | U | N | Y | Y | U | U |
| Hatem Bouraoui (2004) [15] | Y | Y | Y | U | U | N | Y | U | Y | U |
| Vedat Erentug (2004) [16] | Y | Y | Y | U | U | U | U | Y | Y | U |
| Fei Yan (2014) [17] | Y | U | U | Y | Y | N | Y | Y | Y | U |
| Niyazi Gormus (2003) [18] | Y | Y | Y | Y | Y | Y | U | Y | Y | U |
| Vivek Wadhawa (2018) [19] | Y | Y | Y | U | U | Y | Y | Y | Y | U |
| Marah Jamli (2020) [20] | Y | Y | Y | U | U | U | Y | Y | Y | Y |
| Sami S Kabbani (2007) [21] | Y | Y | Y | Y | Y | U | Y | Y | Y | U |
| Mehmet Kaplan (2000) [22] | Y | Y | Y | U | U | Y | Y | Y | Y | U |
| F. Kardaras (1995) [23] | Y | N | N | U | U | Y | Y | U | U | Y |
| A. Miralles (1994) [24] | Y | Y | Y | U | U | U | Y | Y | Y | U |
| Vakeli Murat (2007) [25] | Y | N | N | U | U | U | N | Y | U | U |
| Omer Tanyeli (2017) [26] | Y | Y | Y | U | U | U | Y | Y | U | U |
| Kutay Tasdemir (2000) [27] | Y | Y | Y | U | U | Y | Y | Y | U | U |
| Ertan Onursal (2001) [28] | Y | Y | Y | U | U | Y | Y | Y | U | U |
| Kutay Tasdemir (2009) [29] | Y | Y | Y | U | U | Y | Y | Y | Y | U |
| Haitham Noaman (2017) [30] | Y | Y | Y | Y | Y | U | Y | U | U | U |
| Jose M. Oliver (1988) [31] | Y | Y | Y | U | U | Y | Y | U | U | U |
| Ashur Y. Oraha (2018) [32] | Y | Y | Y | U | U | Y | Y | Y | Y | U |
| A.Abid (2003) [33] | Y | Y | Y | U | U | Y | Y | Y | U | U |
| Özge Altaş (2014) [34] | Y | Y | Y | Y | Y | U | Y | Y | Y | U |
| Dursun Atilgan (2002) [35] | Y | Y | Y | U | U | Y | Y | Y | N | U |
| John Barbetseas (2005) [36] | Y | Y | Y | U | U | Y | Y | Y | U | U |
| Nazim Kankilic (2019) [37] | Y | Y | Y | U | U | Y | Y | Y | U | Y |

**Y: Yes; N: No; U: Unclear.**

***Q1: Were there clear criteria for inclusion in the case series?
Q2: Was the condition measured in a standard, reliable way for all participants included in the case series?
Q3: Were valid methods used for identification of the condition for all participants included in the case series?
Q4:*** ***Did the case series have consecutive inclusion of participants?
Q5:*** ***Did the case series have complete inclusion of participants?
Q6:*** ***Was there clear reporting of the demographics of the participants in the study?
Q7:*** ***Was there clear reporting of clinical information of the participants?
Q8:*** ***Were the outcomes or follow up results of cases clearly reported?
Q9:*** ***Was there clear reporting of the presenting site(s)/clinic(s) demographic information?
Q10:*** ***Was statistical analysis appropriate?***

1. Yasim, A., et al., *Cardiac Echinococcosis: A Single-Centre Study with 25 Patients.* Heart, Lung and Circulation, 2017. **26**(2): p. 157-163.

2. von Sinner, W.N., *CT and MRI findings of cardiac echinococcosis.* European Radiology, 1995. **5**(1): p. 66-73.

3. Tufekcioglu, O., et al., *Echocardiography findings in 16 cases of cardiac echinococcosis: proposal for a new classification system.* J Am Soc Echocardiogr, 2007. **20**(7): p. 895-904.

4. Gürbüz, A., et al., *Cardiac involvement of hydatid disease.* Jpn J Thorac Cardiovasc Surg, 2003. **51**(11): p. 594-8.

5. Birincioğlu, C.L., et al., *Off-pump technique for the treatment of ventricular myocardial echinococcosis.* Ann Thorac Surg, 2003. **75**(4): p. 1232-7.

6. Díaz-Menéndez, M., et al., *Management and Outcome of Cardiac and Endovascular Cystic Echinococcosis.* PLOS Neglected Tropical Diseases, 2012. **6**(1): p. e1437.

7. Akar, R., et al., *Surgery for cardiac hydatid disease: an Anatolian experience.* Anadolu Kardiyol Derg, 2003. **3**(3): p. 238-44.

8. Shehatha, J., et al., *Surgical management of cardiac hydatidosis.* Tex Heart Inst J, 2009. **36**(1): p. 72-3.

9. Tuncer, E., et al., *Surgical treatment of cardiac hydatid disease in 13 patients.* Tex Heart Inst J, 2010. **37**(2): p. 189-93.

10. Abid, A., A. Khayati, and N. Zargouni, *Hydatid cyst of the heart and pericardium.* Int J Cardiol, 1991. **32**(1): p. 108-9.

11. Djoshibaev, S., et al., *Surgical treatment of isolated cardiac echinococciasis: report of five cases.* Anadolu Kardiyol Derg, 2003. **3**(2): p. 137-43.

12. Bashour, T.T., et al., *Echinococcosis of the heart: clinical and echocardiographic features in 19 patients.* Am Heart J, 1996. **132**(5): p. 1028-30.

13. Ben-Hamda, K., et al., *Eighteen-year experience with echinococcosus of the heart: clinical and echocardiographic features in 14 patients.* Int J Cardiol, 2003. **91**(2-3): p. 145-51.

14. Birincioglu, C.L., et al., *Cardiac echinococcosis.* Asian Cardiovasc Thorac Ann, 2013. **21**(5): p. 558-65.

15. Bouraoui, H., et al., *Echinococcosis of the heart: clinical and echocardiographic features in 12 patients.* Acta Cardiol, 2005. **60**(1): p. 39-41.

16. Erentuğ, V., et al., *Cardiac hydatid cysts: surgical treatment and results.* J Card Surg, 2004. **19**(4): p. 358-60.

17. Yan, F., et al., *Surgical treatment and outcome of cardiac cystic echinococcosis.* Eur J Cardiothorac Surg, 2015. **47**(6): p. 1053-8.

18. Gormus, N., et al., *The clinical and surgical features of right-sided intracardiac masses due to echinococcosis.* Heart Vessels, 2004. **19**(3): p. 121-4.

19. Wadhawa, V., et al., *Surgical overview of cardiac echinococcosis: a rare entity.* Interactive CardioVascular and Thoracic Surgery, 2018. **27**(2): p. 191-197.

20. Jamli, M., et al., *Surgical Management and Outcomes of Cardiac and Great Vessels Echinococcosis: A 16-Year Experience.* Ann Thorac Surg, 2020. **110**(4): p. 1333-1338.

21. Kabbani, S.S., et al., *Surgical experience with cardiac echinococcosis.* Asian Cardiovasc Thorac Ann, 2007. **15**(5): p. 422-6.

22. Kaplan, M., et al., *Cardiac hydatid cysts with intracavitary expansion.* Ann Thorac Surg, 2001. **71**(5): p. 1587-90.

23. Kardaras, F., et al., *Fifteen year surveillance of echinococcal heart disease from a referral hospital in Greece.* Eur Heart J, 1996. **17**(8): p. 1265-70.

24. Miralles, A., et al., *Cardiac echinococcosis. Surgical treatment and results.* J Thorac Cardiovasc Surg, 1994. **107**(1): p. 184-90.

25. Murat, V., et al., *Cardiac and pericardial echinococcosis: report of 15 cases.* Asian Cardiovasc Thorac Ann, 2007. **15**(4): p. 278-9.

26. Tanyeli, O., et al., *New World's old disease: cardiac hydatid disease and surgical principles.* Cardiovasc J Afr, 2017. **28**(5): p. 304-308.

27. Taşdemir, K., et al., *Intracardiac Masses.* Asian Cardiovascular and Thoracic Annals, 2000. **8**(4): p. 378-380.

28. Onursal, E., et al., *Surgical treatment of cardiac echinococcosis: report of eight cases.* Surg Today, 2001. **31**(4): p. 325-30.

29. Tasdemir, K., et al., *Surgical approach to the management of cardiovascular echinococcosis.* J Card Surg, 2009. **24**(3): p. 281-4.

30. Noaman, H., et al., *Hydatid Cyst of the Heart.* Angiology, 2017. **68**(9): p. 765-768.

31. Oliver, J.M., et al., *Two-dimensional echocardiographic features of echinococcosis of the heart and great blood vessels. Clinical and surgical implications.* Circulation, 1988. **78**(2): p. 327-37.

32. Oraha, A.Y., et al., *Cardiac Hydatid cysts; presentation and management. A case series.* Ann Med Surg (Lond), 2018. **30**: p. 18-21.

33. Abid, A., et al., *Intracavitary cardiac hydatid cyst.* Cardiovasc Surg, 2003. **11**(6): p. 521-5.

34. Altaş, O., et al., *Cardiac cystic echinococcosis: Report of three cases.* Asian Pac J Trop Med, 2014. **7**(11): p. 922-4.

35. Atilgan, D., et al., *Role of transesophageal echocardiography in diagnosis and management of cardiac hydatid cyst: report of three cases and review of the literature.* J Am Soc Echocardiogr, 2002. **15**(3): p. 271-4.

36. Barbetseas, J., et al., *Cardiac hydatid cysts: echocardiographic findings.* J Clin Ultrasound, 2005. **33**(4): p. 201-5.

37. Kankilic, N., et al., *Unusual Hydatid Cysts: Cardiac and Pelvic-Ilio femoral Hydatid Cyst Case Reports and Literature Review.* Braz J Cardiovasc Surg, 2020. **35**(4): p. 565-572.
